# Qualitative evaluation of a multidisciplinary master of cancer sciences: impacts on graduates and influencing curricular factors

**DOI:** 10.1186/s12909-024-05744-0

**Published:** 2024-07-08

**Authors:** Julia Lai-Kwon, Robyn Woodward-Kron, David Seignior, Louise Allen, Grant McArthur, Michelle Barrett, David L Kok

**Affiliations:** 1 Victorian Comprehensive Cancer Centre (VCCC) Alliance, Melbourne, Australia; 2 Department of Medical Education, Melbourne Medical School, Faculty of Medicine, Dentistry and Health Sciences, University of Melbourne, Melbourne, Australia; 3 Peter MacCallum Cancer Centre, 300 Grattan St, Melbourne, VIC 3000 Australia; 4 Melbourne School of Professional and Continuing Education, University of Melbourne, Melbourne, Australia; 5 Cancer Science Unit, Department of Clinical Pathology, University of Melbourne, Melbourne, Australia

**Keywords:** Oncology, Medical education, Postgraduate education, Online education, Cancer education, Healthcare professional education, Continuing professional development, Interprofessional learning

## Abstract

**Background:**

Evaluations of continuing professional development programs typically focus on short-term knowledge and skill acquisition. There is a need for more comprehensive program evaluation methods that assess a broader range of impacts and can elicit how and why these outcomes occurred. We conducted a qualitative study to investigate the impacts of a multidisciplinary, online health professional postgraduate degree and to gain insights into the factors that led to these impacts.

**Methods:**

Participants were graduates of the University of Melbourne’s Master of Cancer Sciences who could participate in an online interview. Semi-structured, qualitative interviews were conducted exploring a broad range of impacts, including changes in professional practice and career trajectory since graduation, and how the degree influenced these impacts. Data were analysed inductively.

**Results:**

Fifteen participants (female: 80%, 31–50 years old: 67%) from a range of professions were interviewed. A number of major themes were uncovered. Impacts on career trajectory included expanded career horizons (e.g. increased role diversity and complexity), and increased confidence in their professional identity. Impacts on professional practice included individual improvements in patient care and research, as well as changes in organisational practice. Factors identified as leading to these impacts were: (i) active, interactive and interprofessional learning; (ii) networking, informal mentoring, and role-modelling; and (iii) support at multiple levels.

**Conclusion:**

This study provides preliminary evidence of the positive impact of a Master of Cancer Sciences on graduate career trajectory and professional practice. In addition, the inductive methodology enabled identification of the curricular features (both planned and emergent) that influenced these impacts, facilitating potential transferability of learnings to other teaching programs.

**Supplementary Information:**

The online version contains supplementary material available at 10.1186/s12909-024-05744-0.

## Introduction

Continuing professional development (CPD) enables healthcare professionals to maintain, improve and broaden their knowledge, expertise and competence, as well as to develop the personal and professional qualities required in their profession [1]. Thus, participation in ongoing CPD is required for ongoing practitioner registration in most countries [1, 2]. Postgraduate qualifications are regarded as a meaningful form of CPD because they can contribute to career progression and are often linked with pay scales [3–5]. However, healthcare professionals face several challenges in accessing these, including the expense and logistics of travelling to courses requiring in-person attendance, a lack of time, and varying levels of motivation [6–8]. In response to these issues, there has been a continuing increase in the number of wholly online postgraduate degree offerings for health professionals.

Evaluation is a critical aspect of these programs given the significant resources required to develop them and the individual time and opportunity investment required for learners to undertake them. Such course evaluations have traditionally involved appraisal using the Kirkpatrick–Barr model, which categorises program impacts into four levels: 1- reaction; 2- learning; 3- behavior; and 4- results, with higher levels indicating more meaningful impacts [9, 10]. However, quantitative evaluations have difficulty eliciting high-level outcomes, and there have been growing calls for this to be augmented with broader forms of evaluation that can elicit Kirkpatrick-Barr level 3 and 4 outcomes and that can also explain how and why these impacts occurred [11–13]. This knowledge could be used for program quality improvement and would facilitate the transferability of this knowledge to other educational programs.

In light of this, we established an evaluation process for a wholly online Masters degree in Cancer Sciences at our institution. The results of the program’s quantitative evaluation have been previously detailed, with students widely reporting high levels of satisfaction across all areas surveyed [14]. This research builds on these findings by describing the complementary qualitative evaluation.

Our primary aim was to assess the impact of a Masters degree in cancer science on graduates’ career trajectories and professional practices. The secondary aim was to identify some of the pedagogical processes (both planned and emergent) that led to these outcomes.

## Methods

We conducted a qualitative study using semi-structured interviews. A preselected theory was not used to inform the design of the research. Rather, we used a theory-informing inductive approach, which we considered most appropriate given previous issues raised with evaluation in health profession education, specifically, the tendency to focus on predetermined impacts resulting in a neglect of unintended impacts [15].

### Context

This study uses the University of Melbourne/Victorian Comprehensive Cancer Centre (VCCC) Alliance Master of Cancer Sciences degree as a case example of an innovative, wholly online postgraduate degree for health professionals. The course’s design and theoretical and pedagogical underpinnings have been previously described [14]. Briefly, the overall design of the curriculum was guided by the seven principles of online learning [16]. Various pedagogies have also been developed to address the specific needs of cancer professionals, including interprofessionality [17], cognitive load theory [18], cognitive theory of multimedia learning [19–21] and visual information design theory [18].

### Participants and recruitment

Participants were graduates of the Master of Cancer Sciences (2020–2022) program. From September–December 2022, we interviewed 15 participants out of a total of 54 potential graduates (a detailed description of the wider student cohort is described in Lai-Kwon et al. [14]). Purposive sampling was used to ensure that a range of viewpoints were represented, including ages, genders, and current occupations. Potential participants were approached via email from alumni records by a member of the VCCC education team (separate to the research team).

All participants were recruited via purposive sampling. None of the participants were identified via question 9 of the interview (Supplementary Text 1). No additional comments or questions were received in response to question 10 of the interview (Supplementary Text 1).

Table 1 outlines the participants’ demographic information. Most participants were female (*n* = 12, 80%) and aged between 31 and 40 or 41–50 years (each *n* = 5, 33%). The most common professions at the time of enrolment were pharmacists, medical practitioners, and clinical trial assistants employed by clinical research organisations (each *n* = 3, 20%). Ten (67%) participants were > 1 year post graduation from the Masters degree.

**Table 1:** Participant demographics

		*n*	%
Sex	Male	3	20%
	Female	12	80%
Age group	21–30	4	27%
	31–40	5	33%
	41–50	5	33%
	51–60	1	7%
Year of graduation	2020	6	40%
	2021	4	27%
	2022	5	33%
Primary profession at time of enrolment in the Masters	Doctor	3	20%
	Clinical research monitor/assistant (industry)	3	20%
	Nurse	2	13%
	Allied health professional	2	13%
	Pharmacist	2	13%
	Clinical trials assistant (hospital)	1	7%
	Basic scientist	1	7%
	Other	1	7%
Primary profession at time of interview	Clinical researcher	4	27%
	Doctor	3	20%
	Clinical research monitor/assistant (industry)	3	13%
	Allied health professional	2	13%
	Pharmacist	2	13%
	PhD candidate	1	7%

### Ethics and consent process

All potential graduates were sent a plain language statement explaining the study and clearly describing their participation as voluntary. We then obtained informed consent from graduates who elected to participate in the research study. The data were deidentified and stored only by a standardised numbering system. Participant coding was kept by a single investigator and not available to investigators who were academic faculty of the Masters. In addition, participant codes and consent forms were not co-located to reduce the risk of identifying an individual participant. The data were password protected and stored on a secure university server.

The methodology for the study was approved by the University of Melbourne Office of Research Ethics and Integrity (HREC reference 2022-24798-31436-3). As this study is not a clinical trial, a clinical trial number was not required.

### Data collection

Semi-structured interviews were chosen to enable exploration of a range of impacts and to uncover insights into the factors that influenced these impacts. The interview guide was designed to collect baseline demographic data, including age, gender, year of graduation from the Masters degree, occupation prior to enrolment in the Masters degree, and current occupation at the time of the interview, as well as information on how the Masters degree impacted their career trajectory and professional practice (Supplementary Text 1). Barr’s Modification of Kirkpatrick’s Typology of Educational Outcomes [10] was used to inform the development of the interview guide, particularly the inclusion of examples about impact on professional practice, including level 2a (modification of perceptions and attitudes), level 2b (acquisition of knowledge and skills), level 3 (behavioural change), level 4a (change in organisational practice) and level 4b (direct benefits to patients).

The interviews were conducted by JLK or RWK and lasted approximately one hour. The interviews were conducted on Zoom (Zoom, California, United States), and audio and video were recorded. The interviews were automatically transcribed using Otter.AI (Otter.AI, California, United States) within Zoom. Transcripts were then checked for accuracy against audio and visual recordings by JLK and deidentified. Information power was used to determine the sufficiency of the sample [22]. The interviews were carried out until sufficient information power was reached (*n* = 15), which was determined through consideration of the aim, sample specificity, use of theory, quality of dialogue and analysis strategy.

### Data analysis

We used reflexive thematic analysis to analyse the interview data. This type of thematic analysis was chosen because it is a flexible method that allows for a theory-informing, data-driven inductive approach [23, 24]. Our analysis was informed by a social constructivist worldview. This worldview sees reality as being socially constructed and influenced by our backgrounds, experiences, and interactions. Acknowledging this, our research involves the co-creation of knowledge through interactions between researchers and participants [25].

The transcripts were then deidentified, and the first three (20%) transcripts were read line by line by JLK and DS to familiarise themselves with the dataset. JLK and DS coded the transcripts independently using an inductive approach, reflecting on the data itself and their experiences in designing the Masters. This process was iterative, and the findings were discussed and compared regularly. Once the codes agreed, JLK and DS coded the first three transcripts, and any discrepancies were resolved by discussion with the coauthors. The remainder of the transcripts were then coded by JLK. DS also reviewed all codes generated by JLK, and any disagreements were resolved by discussion with the coauthors. Coding was undertaken in NVivo (release 1.7, QSR International, Melbourne, Australia). Codes were initially grouped into the two overarching aims of the study (impact on career trajectory, impact on professional practice). We then undertook a mind-mapping process, following the principles outlined by Buzan and Buzan [26], whereby we visually mapped quotes with similar content into themes, represented as regions, with proximity indicating similarity of theme and lines drawn to show the interconnecting relationships between themes and subthemes. All the researchers were involved in reviewing the themes and subthemes, and final interpretations were achieved by consensus.

**Fig. 1:**
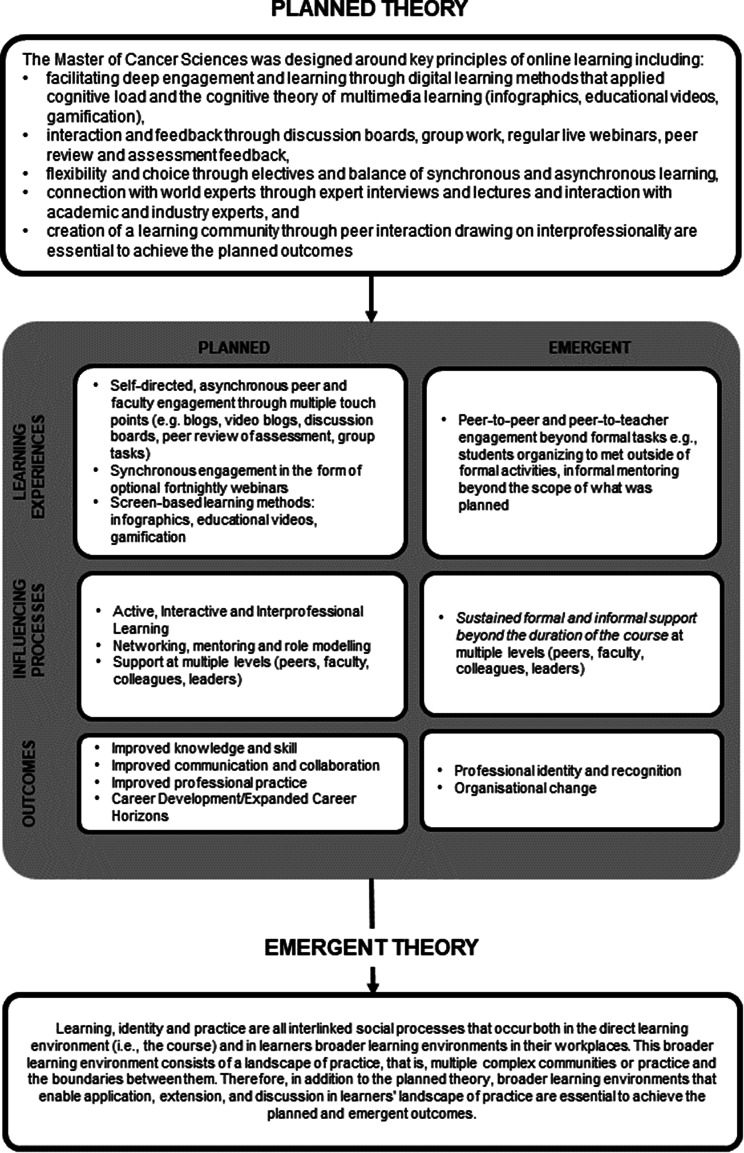
Planned and emergent experiences, processes and outcomes of the Master of Cancer Sciences

As we developed our interpretations of the themes, we selected the Haji et al. model to help illuminate our findings, particularly how and why impacts are occurring [13]. The Haji et al. model articulates planned theory (why the program will work), planned processes (how the program should operate) and planned outcomes (did the intended effects occur). In addition, it also considers emergent processes (what other ways did the program operate), planned outcomes (what other effects did the program have), and emergent theory (why are the planned and emergent outcomes happening), as shown in Fig. 1.

### Reflexivity

We acknowledged our experience as course convenors (DK), learning designers (DS) and faculty (JLK) within the Master of Cancer Sciences informed our inquiry. Delivery of the program enabled the team to observe differences between the planned processes and the emergent processes.

### Rigour

This study was designed to be an authentic evaluation of program impacts; hence, efforts were made to ensure its credibility and authenticity. The interviews were performed by two members of the research team from a structured interview template. One of these was an independent researcher with no involvement in the design or delivery of the course, and the other was a member of the course faculty who was not part of the leadership team. The interviews were conducted to the point of data saturation (i.e., no further themes were elicited in interviews that had not been captured already), and the data analysis and coding were performed by two independent investigators.

## Results

The primary aim of this study was to assess the impact of the Master of Cancer Science degree on graduates’ career trajectories and professional practices. The secondary aim was to identify some of the pedagogical processes (both planned and emergent) that led to these outcomes. In this section, we list the impacts uncovered and the processes that were identified as contributing to these impacts.

Four themes emerged through our reflective thematic analysis. Two of these aligned with our aim to explore the impact on career trajectory – (i) expanded current and future career horizons and (ii) professional identity and recognition – and two aligned with our aim to explore the impact of professional practice – (iii) improved professional practice and (iv) organisational change.

### Expanded current and future career horizons

Participants described how the course had resulted in an expansion of their career horizons, both in current roles and in the roles that they aspired to in the future. This is a Kirkpatrick-Barr level 3 outcome – change in behaviour.

In terms of expanding their current horizons, they described the addition of various education, research, mentoring and leadership roles to their current positions. The Masters also enabled them to apply for new roles and promotions. They perceived the attainment of the qualification as increasing their competitiveness for promotion and enabling them to be promoted more quickly. In some, it also initiated consideration of further study of professional degrees (e.g., medicine), as well as research higher degrees:

*“I’ll consider continuing my formal education*,* a PhD or something along those lines…I think prior to doing the Masters… further studies hadn’t really been on my radar”* (P11, male, graduated 2021).

Additionally, through mentee opportunities, students were able to build their research networks, access research projects and begin incorporating research into their work roles. This, along with the practical research skills obtained through the research capstone, enabled them to embark upon research projects following graduation:

*“I’ve…helped him [the student’s supervisor] on a systematic review …I’ve got a few opportunities through…making a network with my supervisor”* (P5, female, graduated 2021).

Networking opportunities with both fellow students and faculty staff of the Masters course broadened their awareness of potential career pathways:

*“It connected me with people who were academic/clinical/research… I think it was a real…opportunity for me to see the careers that people who run the Masters have*,* the course coordinators*,* the subject tutors and things*,* and the cohort that it put me in touch with”* (P12, female, graduated 2020).

Similarly, obtaining practical leadership skills, alongside the opportunity to reflect upon their leadership style, enabled the participants to improve and expand their leadership roles:

“*I was able to do the two leadership subjects and that I feel has definitely helped with in that sort of mentoring leadership role*,* providing feedback”* (P2, male, graduated 2021).

Modelling of potential career pathways, alongside increased knowledge and skill levels, inspired participants to seek out oncology-specific opportunities and consider subspecialisation in oncology:

*“I’ve been making a conscious effort now to really put my hat in the ring for those [oncology] studies… which are usually given to more senior individuals because I’ve got that knowledge from the Masters”* (P8, female, graduated 2022).

### Professional identity and recognition

The participants described how the Masters impacted both how they saw themselves and how they were seen by others. From a reflective point of view, the Masters enabled them to feel valued and increased their confidence in themselves and their professional skill set. This is both a Kirkpatrick–Barr level 1 and level 3 outcome – a reaction and a change in behaviour, respectively.

This supported their career development and professional practice:

*“I think prior to doing that subject*,* I really felt like you’re either a clinician or you’re an academic and then there’s not much crossover. But I think actually doing the Masters part time and doing a research project while working clinically*,* really demonstrated that having a clinician can really help with the research and make it practical*,* make it the right kind of research that’s actually going to be useful to clinicians”* (P5, female, graduated 2021).

Participants reflected on the components of the course that contributed to this. Having a supportive, diverse cohort from a range of backgrounds enabled them to feel valued. As described by P10, this was different from their prior working experience:

*“We grow up in a model that’s been quite hierarchical…coming into the Masters and working with quite a diverse group of people…as a nurse*,* I felt very really valued and an equal”* (P10, female, graduated 2020).

The supportive environment was facilitated through learning activities that involved active engagement with peers. This helped to break down professional silos and enhance collaborative, nonhierarchical relationships:

*Group work was part of the course and every single subject … conversations just naturally flowed and you learned about…each other”* (P12, female, graduated 2020).

From an external perspective, the graduates received recognition from their colleagues and leadership in the form of awards and recommendations to apply for jobs:

*“I was actually recognised by one of my line managers in there as a future leader of our research institute. As part of that*,* they give you a scholarship for you to go on to university and do*,* you know*,* a postgraduate certificate in leadership”* (P7, female, graduated 2022).

“*They came back to me and said “(You should really put your application in for this position”. I don’t think I would have been capable of taking on without what I’ve learned in…the Masters”* (P7, female, graduated 2022).

Graduates suggested that this was because formal postgraduate qualifications such as Masters were viewed favourably by employers as a show of commitment to ongoing professional development.

### Improved professional practice

The Masters impacted participants’ professional practice, including numerous examples of Kirkpatrick-Barr level 4 outcomes - changes in the care of patients. Some participants provided examples of how their deeper understanding and appreciation of the multidisciplinary team facilitated improved multidisciplinary care:

“*Another lady… she was also seeing another local psychologist…*.”

*“[It was beneficial] just being… aware…and sensitive to the… psychosocial and emotional impact… then also being able to give a good handover or helpful amount of information for the psychologist”* (P2, male, graduated 2021).

This also extended to being confident enough to contribute their knowledge as a member of the multidisciplinary team. For example, one graduate provided an example where they shared their knowledge of side effects with other members of the multidisciplinary team to improve the care of an individual patient:

*“When we were learning about CAR-T therapy … I did have a patient on the wards having CAR-T and after their initial dose reacted quite badly to it…I think being able to escalate that to medical staff and nursing staff*,* to kind of let them know that… we were seeing early signs of some neurotoxicity was really useful”* (P5, female, graduated 2021).

Graduates ascribed these changes to the multidisciplinary focus of the course, including participation in mock multidisciplinary meetings and their interactions with their fellow students from a range of professional backgrounds.

In addition, graduates described being more patient-centred at work. They felt that they had learned to be more empathic, compassionate and better communicators, and these skills improved their patient care in numerous settings.

Patient care setting: *“I think it’s more the communicating and the empathy…so when you see the patients… the things that they’re thinking about is completely different to if you went to see another patient from a different ward*” (P9, female, graduated 2020).

Pharmaceutical setting: *“I did a little bit of compassionate access stuff with drugs as well…but I think the Masters also reinforced the patient focus because you got to hear about clinically*,* this is what a patient is going through”* (P8, female, graduated 2022).

Research Setting: *“it had a profound impact on me doing that thesis… I’ve reached out to them about being on our Consumer Advisory Group and reading patient information sheets for us … because they’re just as keen to be involved”* (P7, female, graduated 2022).

### Towards organisational-level change

Graduates also provided examples of how knowledge and skills gained from the Masters had begun to influence organisational priorities (Kirpatrick-Barr Level 4a – changes in organisational practice), including increased engagement in teletrials and consumer engagement in research:

*“My thesis*,* I actually did that on teletrials and decentralised trials*,* and now I’m trying to take on subject matter expert roles in those to try and progress that within the business”* (P8, female, graduated 2022).

Changes in organisational policies and protocols were also described, such as the adaptation of staff education materials based on knowledge gained from the Masters:

*“One of the things that changed from the Masters course was not just the way we have looked at oncology and assessed oncology*,* but also the processes…just the way that*,* you know*,* someone would go through and assess someone”* (P2, male, graduated 2021).

Another participant described how lessons learned about multidisciplinary care resulted in a change in the way multidisciplinary meetings were conducted at their centre:

*“each week there’s a different person that chairs that meeting”;*

*“I think that’s been something … I learned about doing an assignment in the Masters that we’ve managed to translate into practice”* (P5, female, graduated 2021).

### Mapping the planned and emergent experiences, processes and outcomes using the Haji model

The participants in this study described a range of program outcomes, some of which were planned (i.e., aligned with the intended learning outcomes of the course), while others were emergent. These are mapped and listed in detail in Fig. 1.

Alongside the lower-order Kirkpatrick-Barr outcomes of improved knowledge/skill and improved communication/collaboration, we documented improvements in professional practice, the expansion of career horizons, increased professional identity and recognition and examples of organisational-level change.

## Discussion

This study assessed the impact of a Master of Cancer Science degree on graduates’ career trajectories and professional practices and aimed to identify some of the pedagogical processes (both planned and emergent) that led to these outcomes. Four themes emerged through our reflective thematic analysis. Two of these aligned with our aim to explore the impact on career trajectory – (i) expanded current and future career horizons and (ii) professional identity and recognition – and two aligned with our aim to explore the impact of professional practice – (iii) improved professional practice and (iv) towards organisational change. These were driven by intentional curriculum design aspects (which included active interprofessional learning, networking and mentoring). In addition, sustained formal and informal support from peers and faculty beyond the duration of the degree was observed. This was unplanned but also a strong facilitator of outcomes. Thus, this study provides important design considerations for future health professional education programs and an example of how qualitative evaluation can provide a more complete view of a program’s outcomes.

A key strength of our research is that the qualitative approach enabled us to elicit impacts across the full range of Kirkpatrick–Barr outcomes, which our previous quantitative evaluation was unable to do [14]. In particular, it was able to draw out higher level 3 and 4 outcomes describing behaviour change, change in organisational practice, and change in the care of individual patients. Furthermore, we were able to gain insights into how and why the outcomes occur.

We chose to examine our results from the lens of landscapes of practice. While we did use an evaluation model, this was not used from the outset of the evaluation. Rather, it was used to help make sense of the results. We encourage other clinician-led evaluation teams to select an evaluation framework from the outset that allows for articulation of program theory and exploration of how and why outcomes occur and, where possible, engaging education and/or evaluation experts that can help articulate program theory.

### Understanding the results through the lens of the Haji et al [13] model and landscapes of practice theory

To better depict and understand the planned and emergent outcomes identified in our evaluation, we mapped our findings following the Haji model [13], as shown in Fig. 1. The Haji et al. model was a particularly apt framework for our findings, as it shows the link between planned theory, planned processes and planned outcomes. In addition, it also considers emergent processes (what other ways did the program operate), planned outcomes (what other effects did the program have), and emergent theory (why are the planned and emergent outcomes occurring).

In our study, professional identity and recognition were an emergent outcome, but nonetheless an important outcome as professional identity is necessary for success [27]. We ascribe to the view that professional identity development is a social process [28]. This is notable, as an oft-described disadvantage of online learning is social isolation [29, 30]. To mitigate this, this course was intentionally designed to foster engagement and interprofessional interaction. Indeed, when interrogating the data and reflecting on the emergent processes DK, DS and JLK felt that the level of engagement between students and faculty was higher than expected and frequently went beyond formal course activities.

The participants attributed planned and emergent outcomes to several influencing processes, namely, (i) active, interactive and interprofessional learning; (ii) networking, informal mentoring, and role-modelling; and (iii) support at multiple levels (peers, faculty, colleagues, and leaders). Active, interactive and interprofessional learning were key components of the planned theory addressed by the principles of facilitating deep engagement and interaction and feedback. They promote engagement, interaction and sharing of diverse perspectives, which have been shown to enhance learning [31, 32]. Similarly, networking, mentoring, and role-modelling were key components of the planned theory addressed by the principles of connection with experts and creation of a learning community. Networking, mentoring, and role-modelling offered the participants a chance to learn from and with others, learning and developing their identities through a social process that has been shown to influence personal and career development [33, 34].

From the participants’ accounts, it is clear that both the learning environment of the course and the broader learning environments of their workplaces contributed to the outcomes. Therefore, the third influencing factor, support at multiple levels, was only partially addressed by the planned theory. The creation of a learning community addresses support in the immediate context (that of the online degree) but does not address the learning environment beyond this, with participants describing supportive colleagues and leaders as contributing to their learning and identity development. This finding resonates with Aitken’s research that used activity theory to examine the value and perceived impact of online healthcare postgraduate programmes [35]. Activity theory is a philosophical framework that tries to understand the entire system in which a phenomenon occurs. Hence, rather than just looking at the subject (in this example, students), the object (the learning outcomes) and its mediating artifacts (the online degree), activity theory also studies the complex interactions between these and the context in which they exist [36]. Using this framework, Aitken found that “teaching is delivered online, but learning occurs as the students move through the various contexts they inhabit.” The activity theory perspective acknowledges that there are multiple learning environments and that learning involves influencing others in those environments, building confidence, and expanding thinking and networks. However, it does not consider learning, identity and practice as interlinked social processes as the social learning theory that underpins communities and landscapes of practice does [28, 37].

Communities of practice is a social learning theory that describes learning as participation [28]. Landscapes of practice build on the notion of communities of practice, focusing on the fact that “professional occupations…are constituted by a complex landscape of different communities of practice” [37] and the boundaries between them. Learning occurs through our interactions with this complex landscape, and the boundaries between communities are often an important source of learning. This is because boundaries of communities bring different perspectives together and require the negotiation between communities to determine whether the competence of the other community is relevant. Navigating boundaries can therefore be challenging. A key outcome of learning through the lens of landscapes of practice is knowledgeability – “the complex relationships people establish with respect to a landscape of practice, which make them recognizable as reliable sources of information or legitimate providers of services.” [37](page 23) The course enabled participants to develop knowledgeability of other professions that can help them navigate boundaries. It also contributed to their knowledgeability by helping them to be seen as a reliable source of information by the community. This is important given the changing roles participants reported as an outcome of the course. While the course supported knowledgeability, participants need continued learning and development to fully navigate boundaries and become members of new communities (e.g., a clinician taking on the role of and becoming a researcher). Therefore, our initial emergent theory adds the interlinked social processes of learning, identity and practice, multiple learning environments and landscapes of practice to the planned theory, as also described in Fig. 1.

### Implications for practice and future research

Our research has multiple implications for practice and future research.

First, considering prior research and landscapes of practice theory allows us to see learners as part of a much larger and complex learning system. Given that learning occurs both online as part of the course and in various communities, future online courses should consider how they can support learning across these environments and navigate boundaries [38]. Second, participants described seeing other career paths and others juggling multiple types of practice (e.g., research and clinical) as beneficial. Therefore, placing greater emphasis on this, and other health professional courses on ways to achieve this may be useful. Our emergent theory contributes initial insights into how and why outcomes are occurring. Further research, such as realist synthesis or realist evaluation, could use this initial theory to inform their research to gain further insights into how and why outcomes occur.

From a practical course design perspective, the Master of Cancer Sciences was built with a structured framework to intentionally overcome potential barriers to learning for cancer clinicians. Flexible delivery, cognitive load theory and applied learning design are some of the key methods used to overcome these problems [14]. It was pleasing that this appeared to be effective and resulted in Kirkpatrick-Barr level 3 and 4 outcomes in the cohort. Other educators may consider this same approach when designing (or redesigning) future health professional education curricula.

### Limitations

The limitations of this study include that it was a single time point study. Longer follow-up and/or longitudinal research would allow a better understanding of the influencing factors and how they contribute to learning across learners’ landscapes of practice. This research was conducted in a single context, but the description of the context and use of theory help improve the transferability of the results. Additionally, there is some risk of insider bias in this study, as it was conceived and overseen by members of the Masters faculty. However, attempts were made to minimise this, with one of the two project leaders being completely unrelated to the Masters (RWK), who was also one of the two interviewers for the study. Desirability bias was also possible, with respondents potentially voicing opinions that reflected positively on the experience. Similarly, by simply volunteering for the study, there is a potential that this may have subselected a group of students most favourably impacted by the degree.

## Conclusion

Answering calls to go beyond outcome evaluation and explore how and why outcomes are occurring, we conducted a qualitative evaluation of a Master of Cancer Sciences degree. We found that both planned outcomes (career development and improved knowledge, skill, collaboration, and practice) and emergent outcomes (professional identity development, recognition, and organisational change) occurred. These were driven by intentional curriculum design aspects (which included active interprofessional learning, networking and mentoring). In addition, sustained formal and informal support from peers and faculty beyond the duration of the degree was observed, which was unplanned but also a strong facilitator of outcomes. These features may be worth considering in the design of future health professional programs.

### Electronic supplementary material

Below is the link to the electronic supplementary material.

Supplementary Material 1

## Data Availability

The datasets used and/or analysed during the current study are available from the corresponding author on reasonable request.
