# Supplementary material for: Qualitative evaluation of a multidisciplinary master of cancer sciences: impacts on graduates and influencing curricular factors
Source: BMC Med Educ. 2024 Jul 8;24:734. doi: 10.1186/s12909-024-05744-0 (PMC11229342; doi:10.1186/s12909-024-05744-0)
Supplement: Supplementary file 1 — Supplementary Material 1 [file 12909_2024_5744_MOESM1_ESM.docx]

**Supplementary text 1: Qualitative interview schedule**

A: Baseline demographics

1. What is your name?
2. If you don’t mind me asking- how old are you?
3. What gender do you identify as?
4. What year did you graduate from the Masters of Cancer Sciences?

B: Career trajectory

1. Can you tell me a bit about your work/ study prior to enrolment in the Masters of Cancer Sciences?

*Probes:*

- - What field were you working/ studying in?
  - If working, what were your specific roles and responsibilities?

1. Can you tell me a bit about your work/ study since graduating from the Masters of Cancer Sciences?

*Probes:*

- What field are you working/ studying in?
- If working, what are your specific roles and responsibilities?

1. If moved to the cancer sector following graduation:
   - How did completion of the Masters of Cancer Sciences influence your career move?
   - Did the Masters of Cancer Sciences assist you in obtaining your current role? If so, what specific knowledge and skills were most helpful?
     1. How did activities during the course develop this knowledge/ these skills? Anything particularly inspiring?
2. If remained in the cancer sector following graduation:
   - Did completion of the Masters of Cancer Sciences influence your current role? If so, how?

*Probes:*

- Have you gained additional roles or responsibilities?
- Have you gained additional work-related competencies?
  - Has the knowledge and/or skills gained in the Masters been useful in your current role? If so, what specific knowledge and skills were most helpful?

C: Professional practice

If the participant is currently employed in a role which involves directly caring for people with cancer:

1. Can you describe your current professional scope of practice?
2. How has completion of the Masters affected your current scope of practice?
3. Can you provide an example of the following (*utilising Kirpatrick’s Typology of Educational Outcomes as a framework for examples*):
   - A change the way you thought about a task or situation in your day-to-day practice?
   - New knowledge or skills you have acquired through the Masters?
   - A change in your behaviours at work which was informed by what you learnt in the Masters?
   - Something you changed in your organisation based on what you learnt in the Masters?
   - How you applied some knowledge to caring for a specific patient?

D: Closing questions

1. Would you like to receive a copy of this interview transcript?
2. Is there anyone else who you think would be interested in completing this interview? If so, would you mind giving them my email and asking them to get in touch?
3. Do you have any additional comments or queries?
